# Rethinking MPFL Graft Tensioning: A Conceptual Framework for Anatomy-Driven Dynamic Tensioning Beyond Force and Flexion Angle

**DOI:** 10.3390/medicina62101889

**Published:** 2026-09-29

**Authors:** Jacek Walawski, Oliwer Sygacz, Łukasz Matuszewski, Elizabeth A. Arendt, Marcin K. Waśko

**Affiliations:** 1 Ortosport Lublin, Jana Pawła II 13, 20-535 Lublin, Poland; 2 Allmedica Hospital, Kazimierza Przerwy-Tetmajera 21, 20-362 Lublin, Poland; 3 Doctoral School, Medical University of Lublin, Witolda Chodźki 7, 20-093 Lublin, Poland; oliwer.sygacz@gmail.com; 4 Department of Paediatric Orthopaedics and Rehabilitation, Medical University, 20-093 Lublin, Poland; lukasz.matuszewski@am.lublin.pl; 5 Department of Orthopaedic Surgery, University of Minnesota, Minneapolis, MN 55455, USA; arend001@maroon.tc.umn.edu; 6 The Centre of Postgraduate Medical Education, Department of Radiology, Marymoncka 99/103, 01-813 Warsaw, Poland; marcin@wasko.md; 7 Health Sciences Department, Ignacy Mościcki University of Applied Sciences in Ciechanow, 06-400 Ciechanow, Poland

**Keywords:** MPFL reconstruction, patellar instability, graft tensioning, trochlear dysplasia, patellofemoral joint, biomechanics

## Abstract

Medial patellofemoral ligament (MPFL) reconstruction is widely performed, yet its most critical step—graft tensioning—remains insufficiently defined. Many commonly described techniques rely on predefined force values or fixation angles derived largely from cadaveric studies and may not fully account for variability in patellofemoral anatomy, particularly in dysplastic knees. Persistent instability, stiffness, postoperative range-of-motion limitation, and patellofemoral overload nevertheless remain clinically relevant complications after MPFL reconstruction. This article re-examines the conceptual basis of MPFL graft tensioning and highlights the limitations of force- and/or angle-based approaches. We propose a shift toward anatomy-driven, dynamic, independent graft-arm tensioning, in which graft behavior is guided by trochlear morphology and intraoperative patellar tracking. The proposed objective is to restore physiologic patellar engagement within the trochlear groove across the range of motion rather than adjusting graft tension to reproduce predetermined mechanical parameters derived primarily from experimental studies. By prioritizing patient-specific anatomy and functional patellar tracking, this approach provides a testable conceptual framework for MPFL graft tensioning. No comparative clinical or biomechanical data currently demonstrate that this approach is superior to conventional force- or angle-based strategies. It is presented as a testable hypothesis, and prospective comparative validation is required before it can be recommended in practice.

## 1. Introduction

The medial patellofemoral ligament (MPFL) is the primary passive restraint to lateral patellar translation in early knee flexion [1,2]. It functions in close interaction with dynamic stabilizers, particularly the vastus medialis obliquus, forming a coordinated “guiding system” that directs the patella into the trochlear groove during the initial phase of flexion [1,3,4,5,6]. Disruption of this mechanism following lateral patellar dislocation results in altered patellofemoral kinematics and recurrent instability [1,7,8,9,10,11,12].

MPFL reconstruction has therefore become a cornerstone in the management of patellar instability, with generally favorable clinical outcomes reported across various techniques and graft choices [13,14,15]. Nevertheless, failure rates and complications remain clinically relevant. Persistent instability, anterior knee pain, stiffness, range of motion limitation and patellofemoral overload are reported in a notable proportion of patients, highlighting that restoration of stable and physiological patellofemoral function remains challenging in a subset of patients [13,16].

Two major technical factors have consistently been implicated in these suboptimal outcomes: inaccurate femoral attachment and improper graft tensioning [17,18]. While significant progress has been made in improving femoral tunnel positioning through radiological and anatomical methods, graft tensioning remains poorly standardized and largely dependent on surgeon experience and intraoperative judgment.

Many commonly described approaches to MPFL graft tensioning use either predefined force values (e.g., 2 N, 10 N, or 20 N) or fixation at specific knee-flexion angles, most commonly around 30° [19,20,21]. Although widely described, these strategies are supported predominantly by biomechanical and cadaveric studies, frequently performed in knees without the anatomical abnormalities encountered in patients with patellar instability. Their direct transferability to clinical populations with trochlear dysplasia, patella alta, or abnormal tracking therefore remains uncertain.

Importantly, patellar tracking is not determined solely by soft-tissue constraints but is strongly influenced by osseous morphology, particularly the shape and depth of the trochlea [22]. In dysplastic joints, the patella may fail to engage the trochlear groove appropriately, increasing reliance on soft-tissue stabilizers and altering the functional demands placed on the reconstructed MPFL. Under these conditions, reproducing a predefined graft tension or fixation angle may not necessarily restore physiological biomechanics and may contribute to overconstraint or persistent laxity. Moreover, standardized force-based targets do not explicitly account for interindividual differences in knee size, severity of trochlear dysplasia, or soft-tissue elasticity and resistance to tension.

Furthermore, the native MPFL exhibits non-isometric behavior, with variable tension throughout the arc of motion [16]. This challenges the concept of a universally optimal fixation angle or tension value and provides a biomechanical rationale for evaluating graft behavior dynamically across the range of motion rather than exclusively at a single static position. Nevertheless, many commonly described techniques reduce this complex behavior to reproducible force- or angle-based parameters.

We therefore propose that reliance on isolated mechanical parameters may represent an important limitation of current approaches to MPFL graft tensioning. The aim of this article is to critically examine the limitations of force- and angle-based strategies and to introduce an anatomy-driven, dynamic conceptual framework in which graft tensioning is guided by patient-specific trochlear anatomy and intraoperative patellar tracking. This framework is intended to generate a testable hypothesis for future biomechanical and clinical validation rather than to establish a definitive clinical recommendation.

To support this conceptual analysis, a targeted literature search was performed in PubMed, Scopus, and Web of Science through August 2026 using combinations of the terms “MPFL”, “Medial patellofemoral ligament”, “graft tensioning”, “dynamic tensioning”, “graft force”, and “fixation angle”. Studies were considered relevant if they addressed MPFL graft-tensioning strategies, the biomechanical effects of graft tension or knee-flexion angle at fixation, graft behavior during knee motion, or dynamic approaches to MPFL reconstruction. Additional relevant studies were identified from the reference lists of included publications. Because the purpose of the present work was to provide a conceptual analysis rather than a systematic evidence synthesis, no formal PRISMA-based study selection, risk-of-bias assessment, or quantitative synthesis was performed.

## 2. Limitations of Force-Based Tensioning

Force-based graft tensioning has been widely discussed in MPFL reconstruction, yet recommended values vary substantially across the literature, typically ranging from approximately 2 N to 10 N, with some studies reporting substantially higher forces [19,20,21]. Reported protocols are not always directly comparable, and the reported values are not always unambiguous. Gillis et al., in a cadaveric model evaluating the effect of knee-flexion angle during graft tensioning, state that the graft was tensioned with 300 N, citing the protocol of Melegari et al. [21,23]. In that earlier study, however, 300 N was the quadriceps force applied at 30° of flexion to generate an isometric extensor moment, the graft itself having been tensioned with a 1-kg weight [21]. Whichever value applies to the later study, the discrepancy illustrates how difficult it is to compare tensioning protocols across publications, and it cautions against treating any single published figure as a transferable target. This broad variation highlights the lack of consensus and raises uncertainty regarding the clinical transferability of any single numeric target.

An important limitation of the available evidence supporting force-based approaches lies in its predominantly experimental origin. Most available data are derived from cadaveric studies performed on older specimens with anatomically normal patellofemoral joints. These models may not fully replicate the biomechanical environment of patients with patellar instability, particularly those with trochlear dysplasia, patella alta, or abnormal tracking patterns [24,25]. As a result, the direct transferability of force targets derived from these models to clinical populations remains uncertain.

Following MPFL rupture during the first patellar dislocation, restoration of normal patellar positioning within the trochlear groove may be impaired [26], resulting in a more lateral patellar position in knee extension [27]. Importantly, particularly in the presence of patellofemoral dysplasia, MPFL reconstruction may function not only as a restraint procedure but also as a corrective intervention aimed at re-centering the patella within the trochlear groove. Under these conditions, applying a predefined force derived from cadaver studies of anatomically normal knees may not restore physiological biomechanics and may instead contribute to overconstraint or persistent maltracking [28].

A standardized force-based approach also does not explicitly account for interindividual anatomical variability. Knee size, patellar morphology, and soft-tissue properties differ significantly across patients. Consequently, the same applied force may result in over-medialization in smaller or more compliant joints, while being insufficient to restore stability in larger or stiffer constructs [6]. This limitation may be particularly relevant in pediatric populations and in patients with severe trochlear dysplasia, for whom specifically adjusted cadaveric or in vivo tensioning data are lacking.

Another critical limitation concerns the discrepancy between applied and effective graft tension. Intraoperative application of force—whether manual or instrumented—does not directly translate to the final in situ graft tension. Factors such as tunnel friction, graft passage, fixation method, and implant mechanics may significantly alter the actual load transmitted to the graft [29]. As a result, even accurately applied external forces may not produce equivalent in situ graft loads across different constructs and fixation techniques.

Furthermore, force-based models do not fully reflect the dynamic, non-isometric behavior of the native MPFL [30,31]. The native ligament exhibits variable strain patterns throughout the range of motion [25]. Fixing the graft under a predefined static load at a single flexion angle may therefore fail to capture this complexity and may contribute to non-physiological patellofemoral contact mechanics [16].

Finally, the intraoperative clinical reproducibility of force-based tensioning may also be challenging. The application of low-magnitude forces (e.g., 2 or 10 N) is technically challenging and difficult to standardize intraoperatively. Even with dedicated instrumentation, variability between surgeons and techniques may occur, potentially affecting the reproducibility of this approach.

Taken together, these limitations question whether a single predefined force can serve as a universally applicable endpoint for MPFL reconstruction. A more patient-specific approach may therefore require incorporating trochlear morphology, dynamic patellar tracking, and independent graft-arm behavior alongside quantitative mechanical parameters.

## 3. Limitations of Angle-Based Fixation

Fixation of the MPFL graft at a predefined knee-flexion angle—most commonly 30°—is a widely adopted strategy in clinical practice. This approach is based on the assumption that fixation within early knee flexion allows appropriate graft tensioning while the patella approaches or engages the trochlear groove [25].

However, native MPFL biomechanics are considerably more complex. The ligament demonstrates non-isometric behavior, with length and tension varying throughout the arc of motion rather than remaining constant at a single flexion angle [1,25,32]. Consequently, fixation performed at one specific angle may not ensure appropriate graft behavior across the full range of motion.

In addition, patellar stability is not governed solely by soft-tissue restraints. Engagement of the patella within the trochlear groove begins at approximately 20–30° of knee flexion, after which osseous anatomy becomes the dominant stabilizing factor [33]. This transition challenges the rationale for selecting a single “optimal” fixation angle, as the relative contributions of soft-tissue and bony constraints change continuously during knee motion.

Importantly, the concept of a universal fixation angle does not account for variability in patellofemoral anatomy. In dysplastic joints, delayed or insufficient trochlear engagement alters the biomechanics of patellar tracking, potentially shifting the functional range in which the MPFL contributes to stability [22,34,35]. Under these conditions, fixation at a standard angle such as 30° may not correspond to the critical phase of instability for a given patient.

Furthermore, the literature demonstrates substantial variability in recommended fixation angles, ranging from full extension to as much as 90° of knee flexion [15]. More recently, Gillis et al. found no significant differences in patellofemoral contact pressures when MPFL graft tensioning was performed at knee-flexion angles ranging from 0° to 60° in a cadaveric model [23]. Together, these findings highlight the absence of evidence supporting a universally superior fixation angle.

Another limitation of angle-based fixation is its static nature. Fixing the graft at a predetermined angle assumes that appropriate tension will be maintained throughout the entire range of motion. However, due to the anisometric properties of the MPFL and changes in patellar position and rotation during flexion, graft behavior may vary substantially across the range of motion. As a result, graft arms may become overly tight in certain positions and excessively lax in others, contributing to complications such as restricted motion or residual instability [16].

Finally, angle-based techniques do not incorporate real-time assessment of patellar tracking. By focusing on a predefined joint position rather than dynamic behavior, these methods may fail to ensure proper alignment of the patella within the trochlear groove during functional movement.

Taken together, these limitations suggest that reliance on a single predefined fixation angle may oversimplify a highly dynamic and patient-specific system. A more physiologically relevant approach may require complementing angle-based parameters with dynamic assessment of patellar tracking and patient-specific trochlear morphology.

### Limitations of Double-Bundle and Patella-First Fixation Techniques

The superior and inferior graft arms may exhibit different strain patterns during knee flexion [16,36] Consequently, when both arms are initially fixed to the patella and subjected to equal tension, their subsequent tension behavior may differ throughout the range of motion.

*Anisometry of the native MPFL*

**Figure 1:**
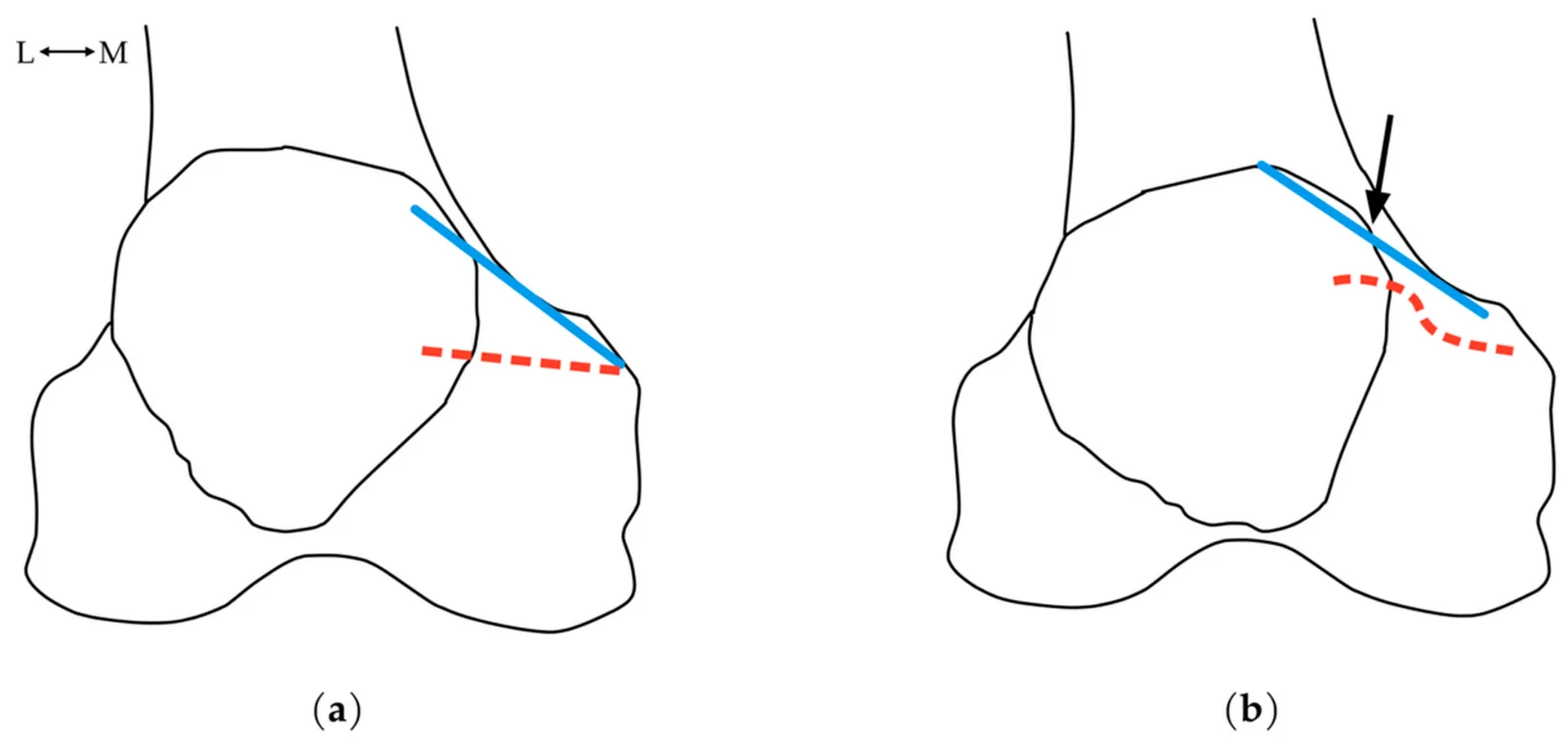
MPFL length change caused by patellar rotation during knee flexion (schematic anterior view of a right knee; not to scale; L, lateral; M, medial). (**a**) At lower degrees of flexion, the upper (blue, solid line) and lower (red, dashed line) bands of the line-shaped MPFL insertion run without crossing and converge towards the femoral attachment. (**b**) As flexion increases, patellar rotation (arrow) alters the orientation of the insertion line, causing the lower band to lose tension (undulating line) while the upper band remains under tension, illustrating the non-isometric behavior of the native MPFL with its line-shaped insertion.

The native MPFL has broad attachment areas at both the patella and femur, with its superior and inferior fibers exhibiting different strain patterns throughout knee motion [26]. However, double-bundle reconstruction does not necessarily reproduce the differential behavior of the native MPFL bundles. As knee flexion increases, the inferior graft arm may lose tension, whereas the distribution of tension within the native MPFL changes throughout the arc of motion [16,32,36]. Excessive tension in the superior graft arm in extension could theoretically contribute to overconstraint and restriction of extension [16,32,36]. This discrepancy may be related, in part, to changes in patellar position and rotation during flexion and suggests that equal initial tensioning of both graft arms—as in some classic V-shaped hamstring, patella-first, double-bundle constructs—may not reproduce the differential strain behavior of the native ligament. These observations provide a biomechanical rationale for considering the differing length–tension behavior of the superior and inferior patellar limbs during MPFL reconstruction [37] (Figure 1).

Importantly, in many commonly described double-bundle techniques, the graft is fixed to the patella before femoral fixation. Once the patellar attachment is secured, the ability to independently adjust the relative tension of the superior and inferior graft arms is substantially limited. This fixation sequence may therefore represent an additional technical factor contributing to differences between reconstructed graft behavior and the differential strain patterns of the native MPFL.

## 4. The Overlooked Role of Trochlear Morphology

Patellar tracking is strongly influenced by the geometry of the femoral trochlea, which becomes a major determinant of patellar engagement and stability during knee flexion. In physiologic conditions, the patella is guided into the trochlear groove early in flexion, providing inherent osseous stability and progressively reducing reliance on soft-tissue restraints [24,38]. This bony guidance mechanism is fundamental to normal patellofemoral kinematics.

In contrast, trochlear dysplasia disrupts this relationship. Flattening or convexity of the trochlear groove, increased lateral inclination, and proximal trochlear deficiency may delay or prevent proper patellar engagement, resulting in persistent lateral maltracking and instability [38,39]. Under these conditions, reliance on medial soft-tissue restraint may increase as osseous containment is reduced or delayed.

This shift has important implications for reconstruction. In dysplastic joints, reproducing native ligament tension derived from anatomically normal knees may not, by itself, be sufficient to restore physiological patellar tracking. From an anatomy-driven perspective, graft tensioning may also need to facilitate appropriate patellar positioning relative to the individual trochlear morphology and engagement within the groove during flexion. Failure to account for this relationship may contribute to persistent maltracking despite technically adequate graft placement and tensioning [40,41].

Moreover, the relationship between trochlear morphology and patellar tracking is dynamic and patient-specific. Variations in trochlear depth, sulcus angle, and lateral facet prominence directly influence the path of the patella throughout the arc of motion [22,24,42,43]. As a result, the applicability of standardized tensioning strategies that do not explicitly incorporate these anatomical factors may vary between patients.

This consideration may be particularly relevant when MPFL reconstruction is combined with trochleoplasty, as surgical modification of trochlear geometry alters the individual osseous relationship guiding patellar engagement [44]. Under these circumstances, predefined force- or angle-based parameters derived from unmodified knees may not fully reflect the postoperative patellofemoral mechanics.

Despite the important role of trochlear morphology, commonly described MPFL graft-tensioning protocols are predominantly based on parameters such as applied force or knee-flexion angle and do not routinely incorporate trochlear morphology as an explicit intraoperative tensioning endpoint.

This represents an important conceptual gap in current approaches to MPFL graft tensioning. Incorporating trochlear morphology alongside dynamic assessment of patellar tracking may provide a more patient-specific framework for intraoperative graft adjustment.

## 5. Toward Anatomy-Driven, Dynamic Tensioning

We propose a shift from force-based and angle-based paradigms toward an anatomy-driven approach, in which graft tension is adjusted according to real-time patellar tracking and trochlear engagement. This concept reframes MPFL reconstruction from a procedure guided primarily by predefined mechanical parameters to one guided by patient-specific anatomy and dynamic patellar tracking.

The surgical technique underlying this approach has been described previously [45]. The purpose of the present article is not to reproduce the technical description, but to examine its conceptual and biomechanical rationale by integrating existing evidence on graft force, fixation angle, differential graft-arm behavior, and trochlear morphology into a testable framework for MPFL graft tensioning.

In this model, the primary objective is to achieve appropriate patellar positioning in early flexion and centered engagement within the trochlear groove as flexion progresses. Rather than applying a predefined force or fixing the graft at a single flexion angle, the surgeon dynamically adjusts graft tension based on intraoperative assessment of patellar position, tilt, and translation during knee motion. This provides an intraoperative means of assessing graft behavior in relation to patellar tracking throughout knee motion rather than relying exclusively on a predefined mechanical target.

Such an approach explicitly acknowledges the non-isometric nature of the MPFL. The native ligament exhibits variable length and tension across the arc of motion, and its functional role changes as the patella transitions from soft-tissue-guided to bone-guided stability. Dynamic tensioning therefore aims to reproduce appropriate behavior across this continuum rather than at a single static reference point.

Importantly, bony anatomy-driven tensioning enables individualized adaptation to each patient’s morphology. Variations in trochlear geometry, patellar morphology, and observed soft-tissue behavior can therefore be considered during intraoperative decision-making. This may be particularly relevant in dysplastic joints, where the timing and quality of trochlear engagement are altered and may not be fully captured by standardized parameters alone.

The proposed framework may also accommodate the altered patellofemoral relationships resulting from concomitant procedures, such as tibial tubercle osteotomy or trochleoplasty, which modify patellofemoral alignment and kinematics [46,47].

From a biomechanical perspective, this concept aligns with the physiological role of the MPFL as a guiding structure rather than a rigid restraint. The ligament primarily facilitates patellar entry into the trochlear groove in early flexion, after which osseous constraints assume the dominant stabilizing function. Accordingly, within the proposed framework, the reconstructed graft is intended to assist in guiding the patella toward appropriate trochlear engagement rather than imposing a fixed position through constant tension.

**Figure 2:**
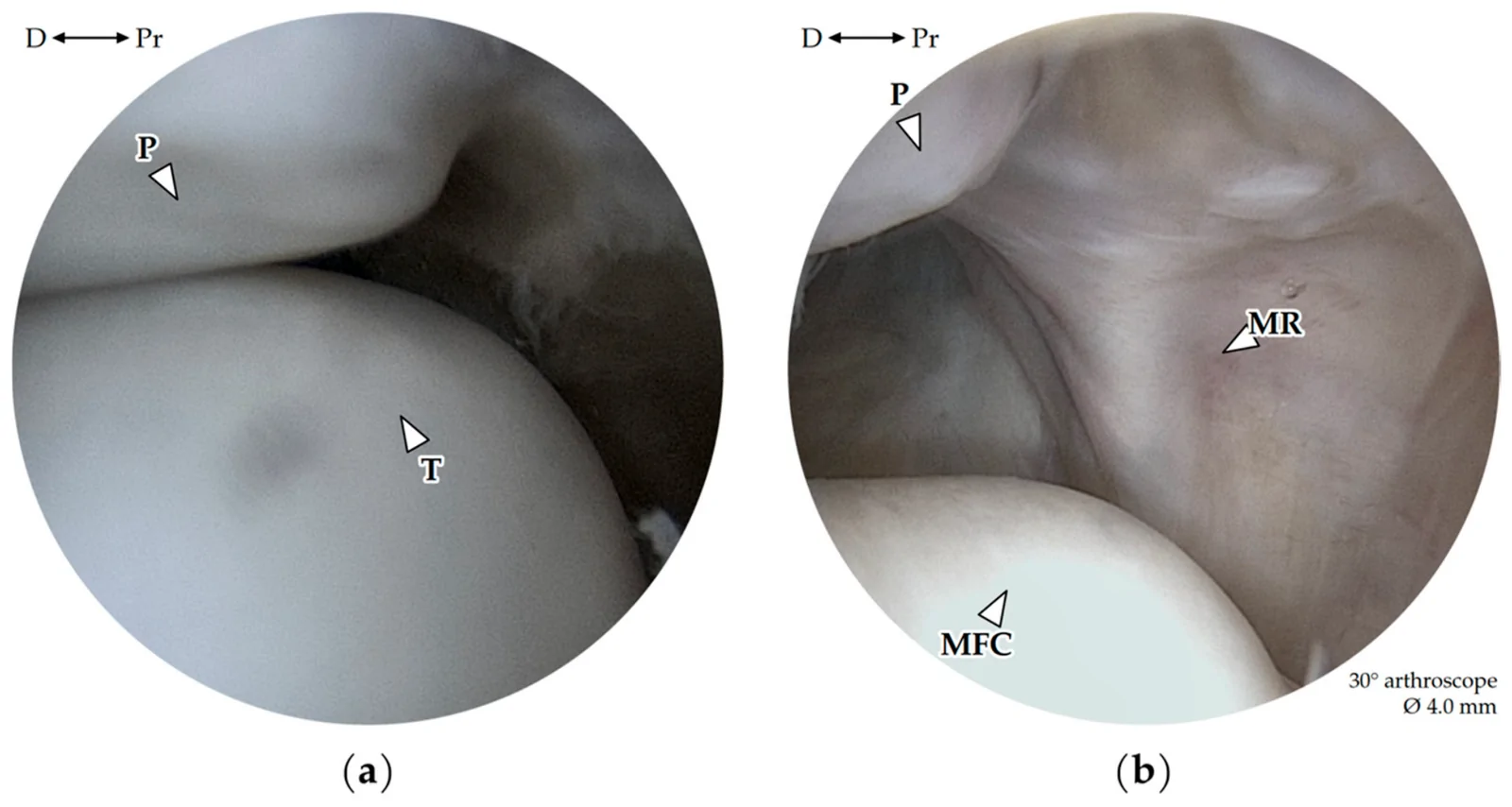
Arthroscopic assessment of patellar repositioning and graft tensioning, viewed from the anterolateral portal with a 4.0 mm, 30° arthroscope at 30° of knee flexion. (**a**) Centralized engagement of the repositioned patella (P) within the trochlear groove (T). (**b**) The MPFL graft (G) used for intraoperative tension assessment. P, patella; T, trochlea; G, graft; MFC, medial femoral condyle; Pr, proximal; D, distal; MR, Medial retinaculum.

Practically, this paradigm encourages intraoperative evaluation of patellar tracking through direct visualization, arthroscopy, or combined methods, with attention to patellar position, tilt, translation, trochlear engagement, and unrestricted knee motion. Rather than defining adequacy by a single numeric force or fixation angle, the proposed functional endpoint is smooth patellar tracking with appropriate trochlear engagement, without evident overconstraint or residual instability (Figure 2). These intraoperative assessment criteria have been applied as part of our previously reported technique [45]. However, their interobserver reliability and the clinical validity of the proposed threshold values have not yet been formally established. At present, no universally validated intraoperative criteria for determining optimal MPFL graft tensioning and physiological patellar tracking are available.

Taken together, anatomy-driven, dynamic tensioning provides a coherent framework that integrates soft-tissue reconstruction with osseous anatomy and joint kinematics. Its potential to improve the reproducibility of graft tensioning and reduce technique-related complications remains to be established through prospective clinical and biomechanical validation.

### 5.1. Patient Selection

In our clinical practice, the proposed technique is used in patients with recurrent lateral patellar instability, defined by a history of two or more patellar dislocation episodes and a positive apprehension test. Patellar instability is additionally reassessed clinically after induction of anesthesia. The technique should not be considered in isolation from the underlying osseous anatomy; major abnormalities that prevent stable patellar engagement within the trochlea require appropriate correction and may limit the applicability of isolated soft-tissue stabilization.

### 5.2. Surgical Technique

**Figure 3:**
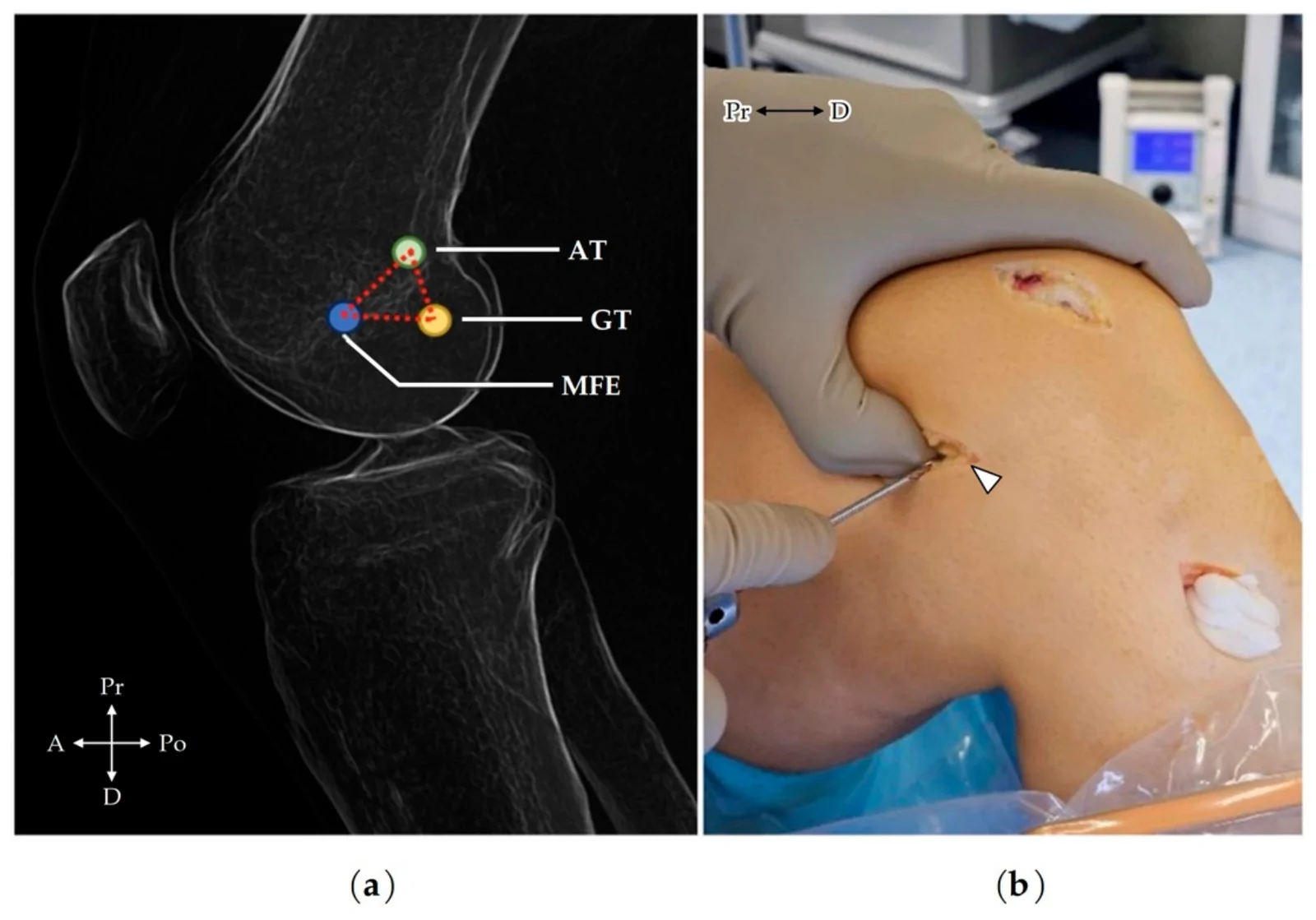
Localization of the femoral insertion point using the Aframian technique [48]. (**a**) True lateral fluoroscopic projection with a schematic overlay of the reference landmarks; the dotted red triangle delineates the target area for the femoral insertion point. (**b**) Corresponding intraoperative view showing identification of the femoral insertion point (arrowhead). AT, adductor tubercle; GT, gastrocnemius tubercle; MFE, medial femoral epicondyle; Pr, proximal; D, distal; A, anterior; Po, posterior.

When indicated, concomitant bony procedures, such as trochleoplasty or tibial tubercle osteotomy, are performed before MPFL reconstruction. In our previously described technique [45], the femoral insertion point is first identified according to Aframian et al. [48], and the isometry of the femoral attachment is verified according to Steiner et al. [49]. The half-folded graft is then fixed within the femoral socket using sliding knots, ensuring appropriate seating of the graft within the socket (Figure 3).

**Figure 4:**
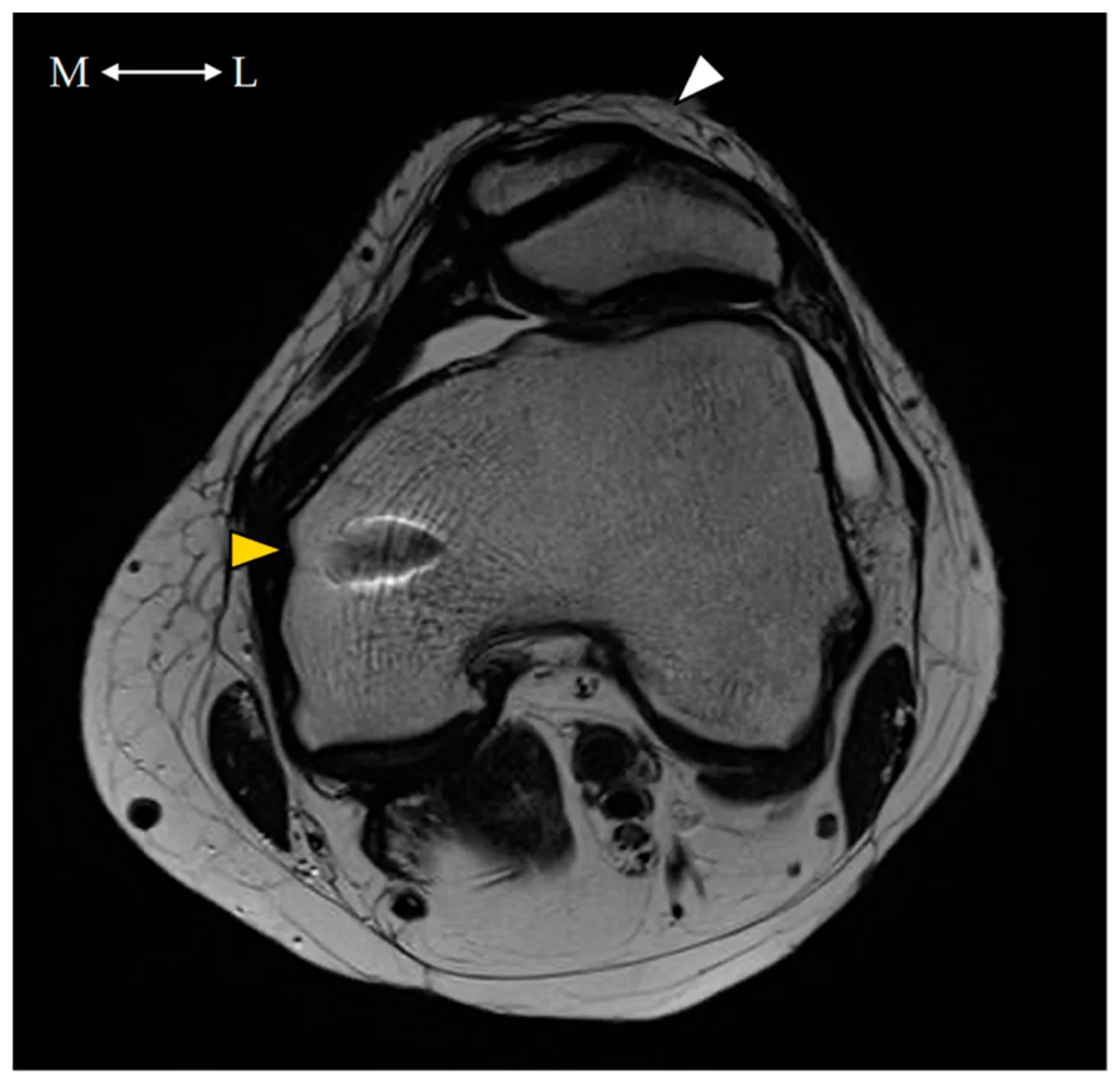
Patellar tunnel drilling, axial proton-density-weighted MR image at the level of the mid-patella. The white arrowhead indicates the patellar graft tunnel outlet; the yellow arrowhead indicates the femoral insertion point of the MPFL graft. Note the difference in their position in the transverse plane. M, medial; L, lateral; MPFL, medial patellofemoral ligament.

Two oblique tunnels, 4.5 mm in diameter, are drilled in the medial proximal half of the patella (Figure 4).

The graft arms are passed through these tunnels and returned to the medial side beneath the patellar retinaculum. No fixation is performed on the patellar side at this stage, allowing independent dynamic adjustment of the two graft arms.

*Over-medialization of the Patella and Graft Tensioning*

**Figure 5:**
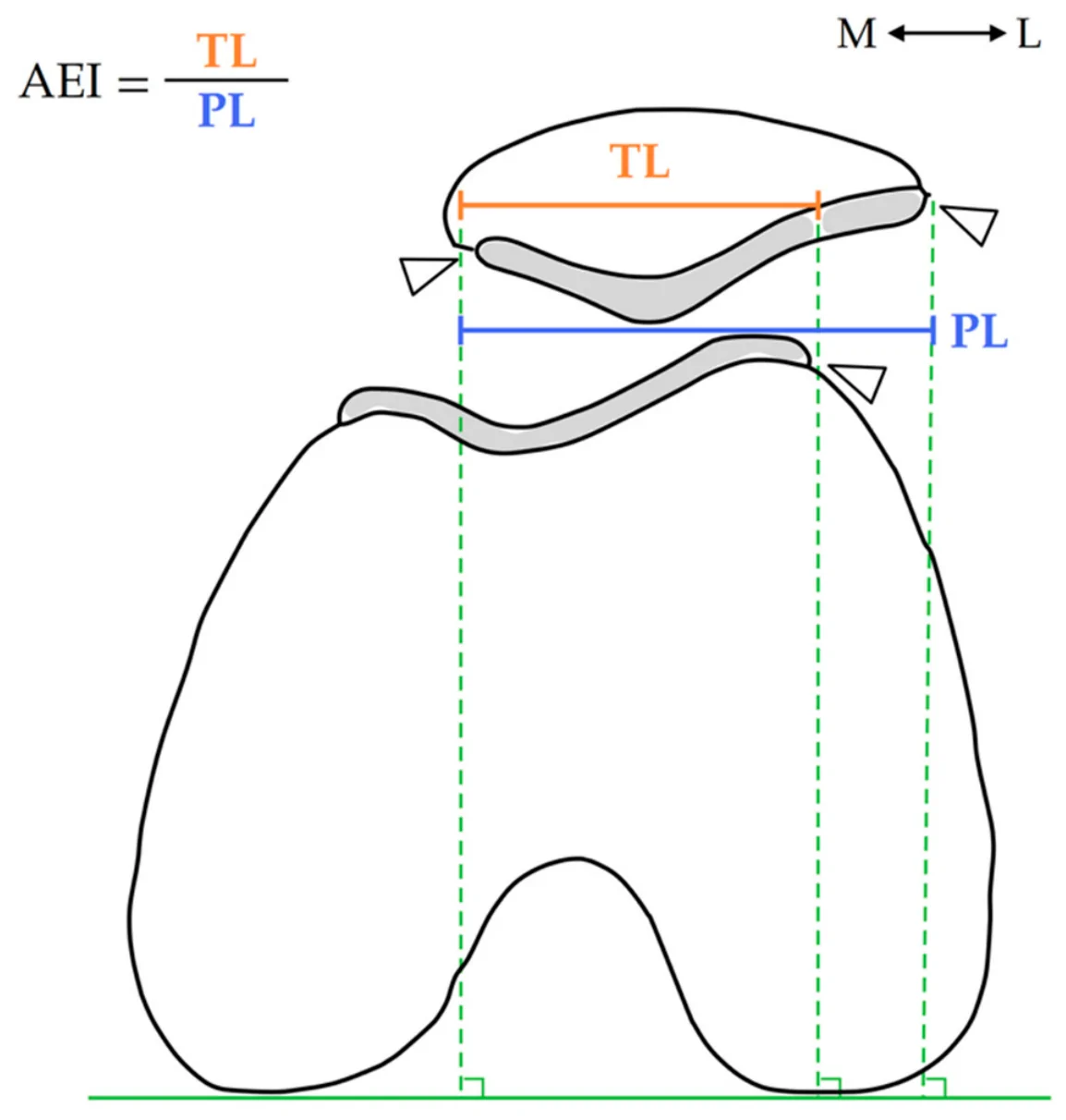
Schematic representation of the patellofemoral axial engagement index (AEI), calculated as the ratio of the projected length of the patellar articular surface engaged within the trochlea (TL, orange) to the projected width of the total patellar articular surface (PL, blue). Arrowheads indicate the reference points: the medial and lateral margins of the patellar articular surface and the lateral trochlear ridge. Green dashed lines indicate the projections from each reference point to the reference plane (solid green line). An AEI approaching 1 indicates near-complete patellar engagement within the trochlear groove. Schematic, not drawn to scale. AEI, axial engagement index; TL, trochlea-engaged length of the patellar articular surface; PL, patellar articular surface width; M, medial; L, lateral.

The lateral margins of the lateral femoral condyle (LFC) and the patella serve as reference points to assess medialization. As a working intraoperative reference, we aim for a patellofemoral axial engagement index (AEI) approaching 1 (Figure 5). It should be emphasised that the AEI was described as a radiological index of patellar engagement [50] and has not been validated as an intraoperative endpoint; the value used here is a pragmatic target rather than a demonstrated threshold, and its reliability is addressed in the Limitations.

**Figure 6:**
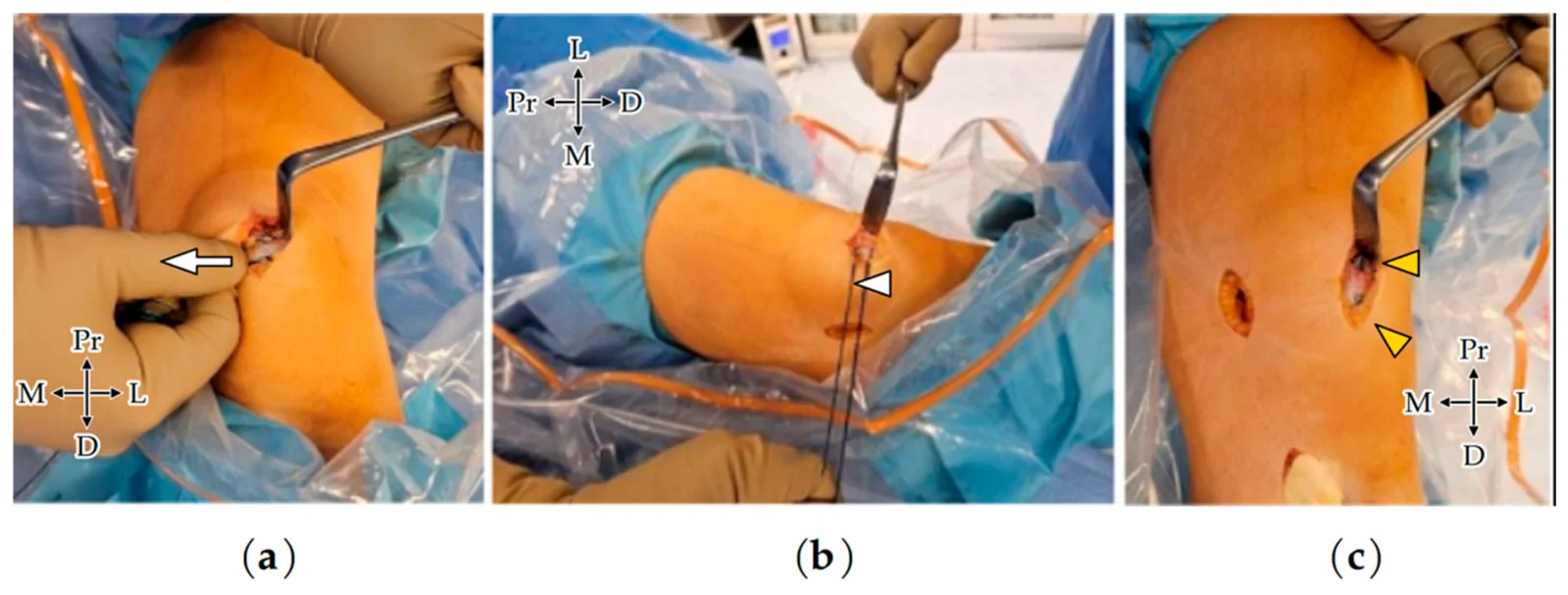
Sequential intraoperative steps of dynamic independent MPFL graft-arm tensioning. (**a**) Temporary over-medialization of the patella in extension (0°), both graft arms remaining unfixed within the patellar tunnels to allow subsequent adjustment; the white arrow indicates the direction of the over-medialization manoeuvre. (**b**) Independent release or re-tensioning of the graft arms during intraoperative assessment of patellar tracking at approximately 30° of flexion; the white arrowhead indicates the graft arm being adjusted. (**c**) Final loop anchorage of the graft arms to the patellar retinaculum (yellow arrowheads) once satisfactory patellar tracking has been achieved throughout the assessed range of motion. Pr, proximal; D, distal; M, medial; L, lateral; MPFL, medial patellofemoral ligament.

The graft arms are intentionally overtensioned to temporarily over-medialize the patella so that, in extension, the patella ‘climbs medially’ onto the initial portion of the medial trochlear facet (Figure 6a). This temporary over-medialization is a controlled and reversible intraoperative maneuver; at this stage, the graft arms are maintained only by manual tension and tunnel friction and remain unfixed on the patellar side, allowing tension to be released or readjusted during subsequent flexion and arthroscopic reassessment [45].

As the knee is flexed, the patella is expected to translate toward the trochlear groove under the guidance of the individual trochlear morphology. The proposed technique uses this anatomic guidance to allow the correction of the temporary over-medialized position during flexion, without the application of an additional medially directed force. If the desired medial position cannot be achieved because of marked lateral restriction, controlled lateral retinacular lengthening may be performed to facilitate medialization. The arthroscope is then reintroduced to reassess patellar tracking and to allow incremental, independent adjustment or release of each graft arm as required (Figure 6b). In our previously described technique, residual lateralization in extension of less than 25% of the patellar width is used as a pragmatic intraoperative criterion [45]. This threshold was selected empirically; neither its interobserver reliability nor its relationship to clinical outcome has been established. Once satisfactory tracking has been obtained throughout the assessed range of motion, final fixation of the graft arms is performed to the patellar retinaculum and/or to the graft itself, creating a “loop anchorage” (Figure 6c).

A detailed step-by-step description of this surgical technique has been previously published in Arthroscopy Techniques [45]. The complete surgical sequence and dynamic intraoperative graft-arm adjustment are demonstrated in Supplementary Video S1.

## 6. Implications for Surgical Practice

The proposed anatomy-driven tensioning strategy has several potential implications for surgical practice, shifting intraoperative decision-making from reliance on predefined parameters toward functional assessment.

First, this approach reinforces the importance of accurate femoral graft placement and isometry as prerequisites for reliable reconstruction. While tensioning strategies may vary, incorrect femoral positioning remains a critical source of failure and cannot be compensated for by the adjustment of graft tension alone [44,46]. Accurate femoral localization therefore provides the anatomical basis for subsequent assessment and adjustment of graft behavior throughout knee motion.

Second, this paradigm recognizes trochlear morphology as a major determinant of patellar tracking. Rather than treating osseous anatomy separately from graft tensioning, the proposed framework uses the relationship between the patella and trochlear groove as an intraoperative reference for graft adjustment. This may be particularly relevant in dysplastic joints, where altered trochlear geometry influences patellar engagement and stability [51].

Third, anatomy-driven tensioning requires intraoperative assessment of patellar position and tracking. This may be performed through direct visualization, dynamic range-of-motion testing, and arthroscopic evaluation of patellar alignment and trochlear engagement, as described in our previous technical report [45]. These assessments provide real-time information on patellar lateralization, tilt, and engagement and allow independent adjustment of graft-arm tension before final fixation.

Fourth, this approach does not rely exclusively on a rigid, predefined tensioning protocol. Instead, graft tension is adjusted dynamically based on patient-specific anatomy and intraoperative findings. This flexibility is particularly important in combined procedures, where additional interventions—such as tibial tubercle osteotomy or soft-tissue releases—may alter patellofemoral mechanics and therefore require reassessment of graft behavior before final fixation.

From a clinical perspective, avoiding both excessive and insufficient graft tension remains an important objective of MPFL reconstruction. Over-tensioning has been associated with increased patellofemoral contact pressures and restricted range of motion, whereas insufficient tension may contribute to persistent instability [32,52]. By incorporating dynamic assessment of patellar tracking before final fixation, the proposed framework is intended to balance these competing risks; whether this translates into fewer clinical complications requires prospective evaluation.

Ultimately, this paradigm promotes a shift toward individualized reconstruction strategies that integrate soft-tissue behavior with osseous anatomy and joint kinematics. Its clinical value relative to conventional force- or angle-based tensioning strategies remains to be established in prospective comparative studies.

## 7. Discussion

We propose a conceptual shift in MPFL reconstruction—from force- and/or angle-directed techniques toward an anatomy-driven, dynamic approach to graft tensioning based on individual trochlear anatomy. The available literature indicates that graft load, fixation angle, femoral attachment, bundle configuration, and trochlear morphology all influence patellofemoral mechanics; however, no single parameter has been established as a universally applicable endpoint for restoring physiological patellar tracking. The proposed approach is therefore consistent with concepts emphasizing restoration of patellofemoral kinematics and contact mechanics, while differing from techniques that rely primarily on a predetermined force or knee-flexion angle as the tensioning endpoint.

### 7.1. Force- and Angle-Based Tensioning in Relation to Previous Evidence

The literature on force-based tensioning is internally inconsistent. In eight fresh-frozen cadaveric knees, Beck et al. reconstructed the MPFL and tested three graft loads—2 N, 10 N and 40 N—measuring medial patellofemoral contact pressure and lateral patellar translation at 30°, 60° and 90° of flexion [19]. Sectioning the MPFL increased lateral translation from 7.7 mm in the intact knee to 16.3 mm. Reconstruction at 2 N restored translation to the intact value without increasing medial contact pressure, whereas 10 N reduced translation to 5.2 mm and 40 N to 1.9 mm, with a significant rise in mean medial contact pressure at 40 N. Two features of this study bear directly on the present argument. First, the transition from physiological restraint to measurable overconstraint occurred between 2 N and 10 N—that is, within the range routinely described in clinical practice as “low tension”—which suggests that the acceptable window is narrow relative to the precision with which tension can be applied, transmitted through a tunnel and maintained during fixation. Second, the variable that discriminated between the tested loads was patellar translation, a tracking measure, rather than the applied load itself. The study therefore supports graft force as a useful experimental input while also showing that the quantity of surgical interest is the resulting patellar position. Its limitations are those of the model: eight anatomically normal specimens, quasi-static loading, no active quadriceps function and no dysplastic knees, so the 2 N value cannot be assumed transferable to a shallow or convex trochlea.

Philippot et al. studied six fresh-frozen cadaveric knees (mean age 69.6 years) using optoelectronic six-degree-of-freedom tracking of the patella through 0° to 90° of flexion under a 10 N quadriceps load [20]. Six acquisitions were made in each specimen: intact, after sectioning the MPFL at its patellar attachment, and after reconstruction tensioned successively to 10, 20, 30 and 40 N. In the intact knee, the patella tilted medially to a mean of 2.02° (SD 3.1), translated medially to 3.3 mm (SD 2.25), and rotated slightly laterally to a maximum of 1.22° (SD 0.8) at 20° of flexion. Of the four tensions tested, only 10 N restored tilt, shift and rotation to values approximating the intact knee; 20, 30 and 40 N produced marked overcorrection of every parameter, and 30 N and 40 N did not differ significantly from one another. The authors concluded that 10 N represents a ceiling rather than a target and recommended intraoperative measurement with a dynamometer. This study matters here for a specific reason: its endpoint was patellar tracking rather than contact pressure, and against that endpoint graft tension proved highly consequential, with the transition to overcorrection falling between 10 N and 20 N.

Dornacher et al. placed six human cadaveric knees in a six-degree-of-freedom simulator and measured mean patellofemoral contact pressure at 0°, 30° and 90° of flexion [53]. A gracilis graft was fixed at 30° of flexion and pre-tensioned to 2, 10 and 20 N, under five reconstruction conditions: anatomical double-bundle, and non-anatomical proximal patellar, distal patellar, proximal femoral and ventral femoral. Raising the pre-tension from 2 N to 20 N did not increase contact pressure at any flexion angle; at 30° the pressure was marginally lower at 10 N (0.71 MPa) than at 2 N (0.77 MPa). Graft position, by contrast, mattered greatly: at 90° of flexion, non-anatomical proximal femoral placement raised mean contact pressure by 257% and ventral femoral placement by 292% relative to the native knee. The authors’ stated hypothesis that higher pre-tension would raise contact pressure was disproved, and they recommended anatomical footprints with a moderate pre-tension of 2–10 N.

Melegari et al. studied 11 cadaveric knees mounted in a loading fixture with the quadriceps tendon clamped, collinear compressive forces being applied to produce isometric extensor moments at 30°, 45°, 60° and 90° of flexion [21]. Their variable of interest was the femoral attachment: an isometric point at the posterior medial epicondyle was compared with a non-isometric point at the adductor tubercle, and patellofemoral contact area and pressure were measured at each angle. Graft tension was held constant throughout, applied by a 1-kg weight chosen because it did not produce excessive tension. With few exceptions, neither the reconstruction itself nor the change from an isometric to a non-isometric femoral point altered contact area or pressure significantly at any angle tested. On this basis, the authors suggested that maintaining graft tension below 1 kg may be desirable—a recommendation arrived at without varying graft tension, as they themselves note in observing that testing other tensions would have provided more information. The study is therefore doubly instructive here. It shows that a construct can satisfy a static mechanical descriptor, isometry, while that descriptor fails to discriminate between attachments with respect to joint loading; and it illustrates how readily a tensioning recommendation enters the literature from a study that did not manipulate tension.

Taken together, these studies do not converge on a transferable numerical target, and in places they are not even mutually legible. Beck et al. identified a narrow tension window in normal cadaveric knees [19]; Melegari et al. showed that a widely used static descriptor does not discriminate between constructs, while recommending a tension they did not vary [21]; and the 300 N figure discussed in Section 2 propagated between two studies with two different meanings. The most instructive comparison, however, is between Philippot et al. and Dornacher et al. [20,53]. Both varied graft pre-tension in cadaveric knees; they reached opposite-looking conclusions because they measured different things. Against patellar tracking, tension was decisive and the window between restoration and overcorrection lay between 10 N and 20 N. Against mean contact pressure, tension from 2 N to 20 N made no difference at all, while femoral tunnel position changed the pressure by up to 292%. Neither result is wrong. They demonstrate that the apparent importance of graft tension depends on the endpoint chosen to judge it—which is the central claim of this article, and the reason we argue that the endpoint should be defined functionally rather than inherited from whichever variable a given rig happens to measure. All of these studies were performed in anatomically normal specimens under quasi-static conditions with little or no active quadriceps function, and none examined dysplastic knees—the joints in which tensioning decisions are most difficult. More recent work is consistent with this reading: Gillis et al. found no difference in patellofemoral contact pressure across fixation angles from 0° to 60° [23], the systematic review by Patel et al. identified no clinically superior fixation angle [15], and recent syntheses of cadaveric tensioning data report that the pressure profile is governed more by femoral tunnel position than by the pre-tension applied within the commonly used range [54,55]. We therefore do not disregard graft force. We read these data as showing that force and angle are relevant biomechanical variables whose appropriate value is patient-specific, and that the endpoint against which that value should be judged is the resulting patellar tracking.

Evidence regarding fixation angle is similarly mixed. Patel et al. found no clear clinical superiority of one fixation angle across the studies included in their systematic review [15], whereas individual biomechanical investigations have evaluated specific fixation angles under controlled experimental conditions [21,23]. More recently, Gillis et al. found no significant differences in patellofemoral contact pressures when MPFL graft tensioning was performed at knee-flexion angles ranging from 0° to 60° in a cadaveric model [23]. These findings are not necessarily contradictory: several fixation angles may produce acceptable biomechanical conditions when the femoral attachment is anatomical, and the graft is not overconstrained. Stephen et al. showed that femoral attachment strongly determines graft length change [25], and Elias and Cosgarea demonstrated that technical errors can overload the medial patellofemoral cartilage [28]. Thus, the present framework interprets the absence of a clinically superior fixation angle not as evidence that angle is irrelevant, but rather as evidence that its effect is context-dependent and influenced by tunnel position, patient anatomy, graft behavior, and patellar engagement.

### 7.2. Dynamic Function and Independent Bundle Behavior

The proposed view of the MPFL as a patellar guide is consistent with foundational biomechanical work showing that the ligament is the principal restraint in early flexion, while trochlear constraint becomes increasingly important after patellar engagement [1,2,6]. It is also supported by Kizher Shajahan et al. and McCulloch et al., who reported angle-dependent strain patterns in native and reconstructed MPFLs [16,36]. Nakagawa et al. further observed that the length-change pattern and shape of a reconstructed tendon differed from those of the healthy ligament even after anatomical reconstruction [22]. Together, these studies indicate that anatomical tunnel placement alone may not be sufficient to reproduce physiological graft behavior.

The present concept extends this evidence by emphasizing independent adjustment of the superior and inferior graft arms throughout the extension–flexion arc. This is supported by Zumbansen et al., who found that selective bundle tensioning improved restoration of dynamic patellofemoral contact pressure compared with nonselective tensioning [56]. Conversely, in patella-first double-bundle constructs, fixation of the graft on the patellar side limits the subsequent ability to independently adjust the relative tension of the two graft arms. The anatomy-driven technique described in this manuscript therefore agrees with the rationale for double-bundle reconstruction [32] but challenges the assumption that equal tensioning of both graft arms can reproduce the differential strain behavior associated with the broad, non-isometric native MPFL attachment. It should be noted, however, that the biomechanical advantage of selective tensioning has not yet been shown to produce superior long-term patient-reported outcomes or lower recurrence rates.

### 7.3. Trochlear Morphology and the Limits of Isolated Soft-Tissue Reconstruction

The central role assigned to trochlear morphology is supported by both computational and clinical evidence. Tanaka et al. and Rezvanifar et al. demonstrated that increasing trochlear dysplasia alters patellofemoral kinematics and promotes lateral maltracking [30,43]. Dejour et al. likewise identified trochlear dysplasia as a major anatomical factor in recurrent instability [38]. These findings support the present contention that force or fixation-angle parameters derived from studies of anatomically normal cadaveric knees may not be directly transferable to dysplastic joints.

Nevertheless, existing clinical evidence also shows that isolated MPFL reconstruction can provide satisfactory outcomes in selected patients with trochlear dysplasia. Steiner et al. reported improvement after reconstruction in this population [49], and broader clinical series and reviews have documented generally favorable outcomes after isolated MPFL reconstruction [13,14]. These observations temper any claim that bony abnormality invariably requires correction. Rather, they suggest that trochlear morphology should inform—not automatically dictate—the procedure. Within the proposed framework, it may be hypothesized that anatomy-driven graft adjustment could be sufficient in selected patients with lower-grade dysplasia, whereas more pronounced anatomical risk factors—including severe trochlear dysplasia, patella alta, major tibial tubercle malalignment, or substantial rotational abnormalities—may favor consideration of combined procedures. The proposed framework is therefore consistent with individualized treatment algorithms and with the ESSKA consensus emphasis on evaluating the complete anatomical risk profile [57,58].

This interpretation is also coherent with evidence that apparently accurate techniques may become less reliable in abnormal anatomy. Izadpanah et al. reported reduced fluoroscopic accuracy of femoral tunnel localization in severe trochlear dysplasia [35], while Nelitz et al. identified technical and anatomical factors in failed reconstructions [39]. Accordingly, intraoperative tracking assessment should complement, rather than replace, accurate femoral localization and preoperative analysis of osseous risk factors.

### 7.4. Clinical Relevance and Areas of Disagreement

The clinical relevance of avoiding both excessive and insufficient graft tension is well supported by the complication profile reported in the literature. Shah et al. documented pain, stiffness, recurrent instability, and other failures after MPFL reconstruction [13], while Stephen et al. and Elias and Cosgarea showed experimentally that reconstruction technique and technical error can increase contact pressure or alter kinematics [17,28]. These data agree with the proposed emphasis on testing tracking throughout the range of motion. However, they do not prove that dynamic tensioning itself reduces complications, because most clinical studies do not report intraoperative graft behavior in a standardized manner and use heterogeneous definitions of tensioning.

Clinical outcome data also show why the restoration of stability should not be equated with complete functional recovery. In our prospective series, patient-reported outcomes improved after patellar stabilization with dynamic independent graft arm tensioning, but quadriceps deficits persisted even at 12 months [59]. This finding is compatible with the present focus on physiological function, yet it also demonstrates that graft mechanics are only one component of recovery; rehabilitation, neuromuscular control and patient compliance also influence the result. Consequently, anatomy-driven tensioning should be regarded as one element of comprehensive patellofemoral treatment rather than a standalone explanation for postoperative function.

A further area of uncertainty concerns reproducibility. Instrumented force targets and predefined fixation angles are attractive precisely because they are measurable. In contrast, temporary patellar over-medialization, trochlea-guided repositioning during flexion, and visual or arthroscopic assessment of patellar tracking retain a partly subjective component. Although specific intraoperative criteria, including patellar engagement and residual lateralization, have been proposed and applied in our previously described technique [45], their interobserver reliability and the clinical validity of the proposed thresholds have not yet been formally established. Stress radiography instrumented patellar mobility assessment, radiological indices, and the axial engagement index may provide additional objective information [40,41,42,50], but none has yet been validated as a universal intraoperative endpoint. The proposed approach therefore replaces reliance on a single numerical target with a more anatomy- and tracking-oriented assessment that still requires further standardization and validation.

### 7.5. Comparison with Existing Tensioning Strategies and the Status of the Evidence

*No direct comparative evidence exists.*

To our knowledge, no randomised trial, prospective comparative cohort or controlled biomechanical experiment has compared anatomy-driven dynamic tensioning with tensioning to a predefined force or at a predefined fixation angle. The framework proposed here is therefore not supported by evidence of superiority and is not offered as a replacement for established protocols (see Table 1). What follows is an account of why we consider the hypothesis worth testing, a comparison with the alternatives, and a specification of the evidence that would be required to accept or reject it.

*Comparison with force-based strategies.*

Force-based tensioning has the substantial advantage of being explicit, measurable and transferable between surgeons and institutions, and it is the strategy for which controlled experimental data exist [19,23]. Its limitation is one visible within those same data: in the only study to test them side by side, the interval between restoring normal lateral translation and measurably restricting it lay between 2 N and 10 N [19]; applied force does not equal in situ graft tension once tunnel friction, graft passage, fixation method and implant mechanics are taken into account [29]; and the reference values derive from anatomically normal specimens. Anatomy-driven tensioning does not offer better measurement. It offers a different endpoint, and the trade-off is explicit: measurability is exchanged for individualisation, and at present only the former has been quantified.

*Comparison with angle-based strategies.*

Angle-based fixation shares the advantage of reproducibility and is simpler still to standardise. The available evidence does not identify a superior angle: Patel et al., in a systematic review of outcomes and complications, found no clinical superiority for any fixation angle [15], and Gillis et al. found no significant difference in patellofemoral contact pressure when tensioning was performed between 0° and 60° in a cadaveric model [23]. We interpret this not as evidence that the fixation angle is irrelevant, but as evidence that its effect is conditional on femoral tunnel position, graft behaviour and osseous anatomy [22,25,28]. That is an argument for context-dependence; it is not an argument for the superiority of the approach proposed here.

*Comparison with isometry-based strategies.*

Selecting the femoral attachment by least length change is an anatomically reasoned alternative to both. It is, however, subject to the same objection: Melegari et al. found no difference in contact area or pressure between isometric and non-isometric femoral points [21], and Nakagawa et al. showed that the length-change pattern of a grafted tendon differs from that of the healthy ligament even after anatomical reconstruction [22]. Isometry constrains the construct less tightly than its intuitive appeal suggests.

*Indirect evidence that motivates the hypothesis.*

Several lines of published work are compatible with the proposed framework without testing it, and we set out each with what it does not establish. Zumbansen et al. found that selective bundle tensioning improved restoration of dynamic patellofemoral contact pressure compared with non-selective tensioning [56]; this is the closest existing biomechanical support for independent graft-arm adjustment, but it was a cadaveric study and has not been linked to any clinical outcome. Kizher Shajahan et al. and McCulloch et al. documented differing strain patterns between the superior and inferior fibres of the native and reconstructed MPFL [16,36], and Nakagawa et al. showed that anatomical tunnel placement alone does not reproduce native graft behaviour [22]; these observations establish that a discrepancy exists, not that adjusting the arms independently corrects it. Izadpanah et al. showed that fluoroscopically guided femoral tunnel placement is inaccurate in severe trochlear dysplasia [35], and Tanaka et al., Rezvanifar et al. and Dejour et al. established that dysplasia alters patellofemoral kinematics [30,38,43]; together, these indicate that static, radiographically referenced targets become least reliable in the population in which tensioning decisions matter most, but they do not show that an intraoperative functional assessment is more reliable there. Each observation is consistent with the hypothesis. None demonstrates it.

*Our own data do not demonstrate superiority.*

We have reported a prospective series in which patient-reported outcomes improved after surgical patellar stabilization with dynamic independent graft-arm tensioning, while quadriceps strength deficits persisted at 12 months [59,60]. That series was uncontrolled and had no comparator arm. It establishes feasibility and acceptable short-term clinical results; it does not establish advantage, and we state this explicitly so that it is not read as supporting evidence.

*What would constitute evidence of superiority?*

Testing this hypothesis requires a prospective comparative study, ideally randomised, in which anatomy-driven dynamic tensioning is compared with a standardised control protocol—for example, fixation at 30° of flexion under 2 N of applied tension—while controlling for radiographically confirmed femoral tunnel position, graft type and diameter, patellar height, tibial tubercle–trochlear groove distance and concomitant procedures, with stratification by Dejour grade of trochlear dysplasia. Appropriate primary endpoints would be recurrent instability or a positive apprehension test at 24 months, or a patient-reported score with an established minimal clinically important difference in this population. Secondary endpoints should include postoperative range of motion, patellofemoral tracking or contact mechanics assessed by dynamic imaging, return to sport, and reoperation. Three methodological endpoints are, in our view, prerequisites rather than secondary aims: the interobserver and intraobserver reliability of the intraoperative assessment, reported as intraclass correlation coefficients; the criterion validity of the AEI and of the 25% residual-lateralization threshold against postoperative imaging and clinical outcome; and the learning curve, quantified for example by cumulative sum analysis of operative time and of assessment agreement. Until such data exist, the framework proposed here should be regarded as a hypothesis with a defined test, not as a recommendation.

### 7.6. Limitations

This article is a conceptual analysis rather than a systematic review, a biomechanical experiment or a comparative clinical study. Its limitations fall into eight groups.

*Methodological limitations.*

The literature underpinning this work was identified through a targeted, narrative search of PubMed, Scopus and Web of Science up to August 2026, restricted to publications in English. Screening and selection were performed by the authors without duplicate independent review; no formal eligibility criteria, risk-of-bias appraisal or quantitative synthesis were applied, and no protocol was registered. The review is therefore open to selection and citation bias, including bias toward studies consistent with the hypothesis advanced here, and the evidence cited should not be regarded as exhaustive.

*Heterogeneity of the underlying evidence.*

The studies discussed differ in specimen age and condition, graft type and diameter, femoral and patellar fixation, applied loads, knee-flexion angle at fixation, loading protocol, measurement system and definition of satisfactory tracking. Reported graft loads span at least 2 N to 40 N in single-bundle cadaveric work [19] and considerably higher fixed loads in more recent protocols [23]. Direct numerical comparison across these studies is therefore not possible, and the synthesis we offer is qualitative.

*Intellectual conflict and circularity.*

The framework proposed here was derived from a surgical technique previously described by the present authors [45] and is illustrated with intraoperative images from that technique; the same group reported the clinical series cited in relation to it [59,60]. We are therefore not independent assessors of the concept, and this article should be read as the authors’ hypothesis rather than as a disinterested appraisal.

*Level of evidence.*

No new clinical, radiological or biomechanical data are reported. No patients were enrolled and no consecutive series is presented; the figures depict single representative cases and are not accompanied by a denominator, a complication rate or an outcome measure. No effect size, confidence interval or comparative statistic can therefore be derived from this work.

*Measurement and validation.*

The intraoperative parameters referred to here—the axial engagement index (AEI) and a threshold of less than 25% residual lateralization of the patellar width in extension—are provisional working references, not validated endpoints. The AEI was originally described as a radiological index of patellar engagement [50]; it has not been validated for intraoperative use, for use in the anaesthetised and unloaded limb, or as a target for graft tensioning. The 25% threshold was selected empirically. For neither parameter have interobserver and intraobserver reliability (for example, intraclass correlation coefficients), the smallest detectable change, or any association with patient-reported or recurrence outcomes been established. They are stated explicitly here so that the proposed assessment is reproducible enough to be tested, not because their accuracy has been demonstrated.

*Limitations intrinsic to the intraoperative assessment.*

Arthroscopic and visual evaluation of patellar position, tilt, translation and trochlear engagement is unblinded and partly subjective, and is performed in an anaesthetised, unloaded limb without active quadriceps contraction. Fluid distension, tourniquet use, limb positioning and residual muscle tone may all influence the tracking observed, which need not correspond to tracking under physiological load. The approach also adds intraoperative time, and the learning curve associated with sequential arthroscopic reassessment and independent graft-arm adjustment has not been quantified.

*Generalizability.*

The described method of independent patellar-sided adjustment presupposes a double-bundle construct in which both graft arms remain unfixed during assessment. It is not directly transferable to single-bundle reconstructions, to quadriceps-tendon techniques such as the superficial quad technique [61], to MPFL repair or imbrication, or to constructs fixed on the patellar side first. It has not been examined in skeletally immature patients, in whom physeal-sparing femoral placement constrains tunnel position, nor in the revision setting. Its applicability is further limited where major uncorrected osseous abnormality prevents stable patellar engagement within the trochlea.

*Absence of comparative evidence.*

Most importantly, no study—clinical or biomechanical—has compared anatomy-driven dynamic tensioning with force-based or angle-based tensioning under controlled conditions. The arguments presented above are mechanistic and indirect and do not establish superiority. Postoperative function additionally depends on rehabilitation, neuromuscular recovery and patient compliance, which this framework does not address and which contributed to the persistent quadriceps deficits observed in our own series despite satisfactory clinical improvement [59,60]. These limitations preclude any causal claim and support interpreting the present framework as hypothesis-generating rather than as an established clinical standard.

### 7.7. Future Perspective: Testing an Anatomy-Driven Strategy

Prospective studies should compare anatomy-driven dynamic tensioning with standardized force- and angle-based techniques while controlling for femoral tunnel position, graft type, trochlear dysplasia, patellar height, tibial tubercle alignment, and concomitant procedures. Relevant outcomes should include recurrence, postoperative range of motion, patient-reported scores, strength recovery, imaging-based tracking, and patellofemoral contact mechanics. Recording the flexion angle at trochlear engagement and the behavior of each graft arm would allow the proposed mechanism to be tested directly. In addition, interobserver reliability of the proposed intraoperative assessment criteria and the clinical validity of the currently proposed thresholds should be evaluated prospectively. The learning curve associated with arthroscopic tracking assessment and independent graft-arm adjustment should also be quantified.

### 7.8. Final Considerations

Taken together, previous studies support the importance of graft tension [19,21,23], fixation angle [15,23,25], femoral tunnel position [17,25,28,35], graft-bundle behaviour [16,22,36,56] and bony morphology [30,38,43,51], but they do not establish any single parameter as a universally applicable endpoint for restoring physiological patellofemoral mechanics. The present analysis integrates these findings by proposing that numerical parameters should be interpreted in the context of the functional endpoint of physiological patellar tracking rather than as isolated targets. This conclusion is consistent with existing biomechanical evidence but remains to be confirmed clinically in prospective studies. Anatomy-driven, dynamic tensioning should therefore be considered a testable framework for personalized MPFL reconstruction, not an established replacement for all conventional protocols.

## 8. Conclusions

Current MPFL reconstruction techniques commonly use predefined graft force or fixation at a specific knee-flexion angle to guide graft tensioning. Although these approaches provide measurable and reproducible intraoperative parameters, the available literature has not established a single force or fixation angle as a universally applicable endpoint across the spectrum of patellofemoral anatomy.

The evidence reviewed in this article indicates that graft tension, fixation angle, femoral attachment, differential graft-arm behavior, and osseous morphology interact in determining patellofemoral mechanics. Because much of the evidence supporting specific tensioning parameters derives from controlled experimental models, their direct transferability to patients with abnormal patellofemoral anatomy, particularly trochlear dysplasia, remains uncertain.

We therefore propose an anatomy-driven, dynamic framework for MPFL graft tensioning in which graft adjustment is guided by individual trochlear morphology and intraoperative assessment of patellar tracking rather than by reliance on a predefined force or fixation angle alone. The intended functional endpoint is appropriate patellar positioning in early flexion and centered trochlear engagement as flexion progresses, while avoiding evident overconstraint or residual instability.

This approach emphasizes individualized reconstruction, integrates soft-tissue and bony factors, and acknowledges the non-isometric and dynamic nature of the MPFL. Rather than replacing force or fixation angle as relevant biomechanical variables, the framework places these parameters within the broader context of patient-specific anatomy and functional patellar tracking.

We wish to state without qualification that no direct comparative clinical or biomechanical evidence currently demonstrates that anatomy-driven dynamic tensioning performs better than force- or angle-based tensioning. The value of this article, if it has one, lies in specifying a mechanism precisely enough that it can be tested and, if necessary, refuted.

Prospective comparative studies, including formal evaluation of intraoperative reproducibility and the clinical validity of the proposed assessment criteria, are required before this framework can be considered an established alternative to conventional tensioning protocols.

## Figures and Tables

**Table 1:** Comparison of intraoperative graft-tensioning strategies in MPFL reconstruction.

Attribute	Force-Based	Angle-Based	Isometry-Based	Anatomy-Driven Dynamic (Proposed)
Intraoperative endpoint	Predefined graft load (e.g., 2 N)	Fixation at a predefined flexion angle (most often 30°)	Femoral point of least graft length change	Patellar position and trochlear engagement through the arc of motion
Principal supporting evidence	Cadaveric [19,23]	Cadaveric and clinical [15,23,25]	Cadaveric [21,49]	Indirect and mechanistic [16,22,36,56]; no comparative study
Reported values or thresholds	2–10 N commonly reported; 40 N overconstrains [19]	0–90° reported; most commonly 30° [15]	Posterior medial epicondyle vs. adductor tubercle [21]	AEI approaching 1; residual lateralization < 25% of patellar width [45,50]—not validated
Measurability and reproducibility	High; explicit and instrumented	High	Moderate	Currently low to moderate; reliability not established
Accounts for applied vs. in situ tension discrepancy	No [29]	Partially	No	Indirectly, through a functional endpoint
Accounts for trochlear dysplasia	No	No	No	By design; not yet demonstrated
Accounts for differential graft-arm behaviour	No	No	No	By design
Evidence of clinical superiority	None established	None established [15]	None established	None established

AEI, axial engagement index; MPFL, medial patellofemoral ligament.

## Data Availability

No new data were created or analyzed in this study. Data sharing is not applicable to this article.

## Supplementary Materials

The following supporting information can be downloaded at: https://www.mdpi.com/article/10.3390/medicina62101889/s1. Video S1: Arthroscopy assisted dynamic MPFL graft tensioning with adaptive length using double bundle technique.

## Abbreviations

The following abbreviations are used in this manuscript:

A	Anterior
AEI	Axial engagement index
AT	Adductor tubercle
CT	Computed tomography
D	Distal
ESSKA	European Society of Sports Traumatology, Knee Surgery and Arthroscopy
GT	Gastrocnemius tubercle
L	Lateral
LFC	Lateral femoral condyle
M	Medial
MFC	Medial femoral condyle
MFE	Medial femoral epicondyle
MPFL	Medial patellofemoral ligament
MR	Medial retinaculum
MRI	Magnetic resonance/magnetic resonance imaging
P	Patella
PL	Patellar articular surface width
Po	Posterior
PRISMA	Preferred Reporting Items for Systematic Reviews and Meta-Analyses
Pr	Proximal
SD	Standard deviation
T	Trochlea
TL	Trochlea-engaged length of the patellar articular surface
